# PD‐1 mRNA expression in peripheral blood mononuclear cells as a biomarker for different stages of primary gouty arthritis

**DOI:** 10.1111/jcmm.15582

**Published:** 2020-07-08

**Authors:** Jing Su, Xuefang Zhang, Qing Zhao, Zhaodi Guo, Jianxiong Wu, Guoqiang Chen, Qianxin Liang, Zhixiang Chen, Zhiliang He, Xiuping Cai, Manlin Xie, Lei Zheng, Kewei Zhao

**Affiliations:** ^1^ Third Affiliated Hospital of Guangzhou University of Chinese Medicine Guangzhou China; ^2^ Guangzhou University of Chinese Medicine Guangzhou China; ^3^ Hai Zhu Qu Jiang Hai Jie Community Health Service Center Guangzhou China

**Keywords:** biomarker, mRNA expression, peripheral blood mononuclear cell, primary gouty arthritis, programmed cell death protein 1, qRT‐PCR

## Abstract

There is currently a lack of biomarkers to assist the diagnosis and prediction of primary gouty arthritis (PG). Therefore, we evaluated the clinical value of programmed cell death protein 1 (PD‐1) mRNA expression in peripheral blood mononuclear cells (PBMCs) of patients with PG. This study included 36 patients with acute phase PG (APPG), 48 with non‐acute phase PG (NAPPG), 42 with asymptomatic hyperuricemia (AH) and 79 normal controls (NCs). PD‐1 mRNA expression levels were detected by qRT‐PCR. PD‐1 mRNA expression was statistically analysed by ANOVA or *t* tests, while correlations between PD‐1 mRNA and clinical variables were assessed using Pearson correlation tests. Receiver operator characteristic (ROC) curve analysis was used to evaluate the diagnostic value of PD‐1 in different PG stages. PD‐1 mRNA expression was significantly lower in patients with APPG than that in NAPPG, AH and NCs (*P* < 0.01). Correlation analysis revealed that PD‐1 mRNA levels correlated negatively with T‐score (*r* = −0.209, *P* < 0.01). ROC curve analysis showed that serum uric acid (SUA), PD‐1 mRNA and both combined displayed higher diagnostic value in patients with PG, NAPPG and APPG compared to that in NCs and patients with non‐PG arthritis (NPG). Moreover, ROC curve analysis showed that SUA and PD‐1 mRNA had good diagnostic value in APPG, with the greatest diagnostic power when combined. PD‐1 mRNA could be a clinical auxiliary diagnostic biomarker for APPG, and the combined use of PD‐1 mRNA and SUA is better than that of SUA alone.

## 1 INTRODUCTION

The most common form of arthritis is primary gouty arthritis (PG), a group of inflammatory diseases in which high serum uric acid (SUA) levels (hyperuricemia) cause joint damage and urate accumulation in the joint capsule, bursa, cartilage, bone and other tissues.^1^ Recent epidemiological studies have shown that the hyperuricemia is widespread, while the prevalence of PG has also increased year by year, reaching 2.49% in developed countries, entering the list of Common and Multiple Global Diseases.^2^, ^3^, ^4^ According to the disease progression, PG is usually divided into acute phase PG (APPG) and non‐acute phase PG (NAPPG) by clinicians. Currently, PG is diagnosed by comprehensively evaluating the overall condition of the patient according to the latest and most authoritative diagnostic criteria for PG, the 2015 American Rheumatology Association or European Association of Rheumatoid Arthritis Classification Standards.^5^ However, to make an accurate treatment plan during clinical diagnosis, the collection of clinical symptoms, blood index tests and imaging examinations during different stages of PG is required. Since the clinical diagnosis of PG can be more complicated at different stages, SUA levels are often used to assist PG diagnosis; however, it is difficult to differentiate between asymptomatic hyperuricemia, NAPPG and APPG using SUA levels since they lack diagnostic specificity.^6^ Therefore, it is necessary to investigate other potential biomarkers in the blood that can assist the differential diagnosis of patients with APPG and NAPPG.

The pathogenesis of PG is complex, and it involves genetic, environmental and immune factors.^7^ Previous studies have suggested that the mechanism includes the deposition of urate crystals in the tissues around joints, which causes mononuclear macrophages to become chemotactic and phagocytose the urate crystals, releasing a variety of pro‐inflammatory cytokines and a large number of inflammatory mediators. These then recruit more mononuclear macrophages to the surrounding tissues where urate crystals are deposited, amplifying the inflammatory cascade.^8^ Recent studies have shown that inflammation and the immune environment are essential factors in the pathogenesis of APPG. During inflammatory immune processes, peripheral blood mononuclear cells (PBMCs) such as T cells, B cells and macrophages infiltrate joint synovial membranes and cells. The interactions between these cells increase pro‐inflammatory cytokine secretion and reduce anti‐inflammatory cytokine secretion, thus exacerbating the onset of inflammation.^9^ T cell activation requires dual signals: the combination of the antigen peptide‐MHC molecule complex and T cell surface receptor (TCR) provides the first signal, while the second results from the interaction between antigen‐presenting cells (APCs) and T cell surface co‐stimulatory molecules, such as the B7/CD28 family and the tumour necrosis factor/tumour necrosis factor receptor (TNF/TNFR) superfamily.^10^

Programmed cell death protein 1 (PD‐1) is a co‐stimulatory molecule belonging to the B7/CD28 family, which are mainly expressed in mononuclear cells and are critical immunosuppressive molecules involved in inflammation.^10^, ^11^ PD‐1 is known to play a negative regulatory role in the immune response and has significant immunosuppressive effects in inflammation.^12^, ^13^, ^14^ Recent studies on the role of PD‐1 in the immune mechanisms, diagnosis and treatment of tumours have shown that it has promising future applications,^15^, ^16^, ^17^, ^18^, ^19^, ^20^, ^21^ while others have demonstrated that it has good auxiliary diagnostic value in rheumatoid arthritis, tumours and other complications.^22^ It has been shown that the level of immune molecules secreted by mononuclear cells are closely correlated with inflammatory immunity in patients with PG,^23^, ^24^ while studies have confirmed that small RNAs play critical regulatory roles in the occurrence and development of APPG.^25^ Although numerous studies have demonstrated that PD‐1 can be widely used for clinical diagnosis and as an auxiliary diagnostic biomarker,^26^, ^27^ the role of PD‐1 in APPG has not yet been studied.

Therefore, we investigated whether PD‐1 is an immunosuppressive molecule secreted by monocytes, which is involved in APPG pathogenesis, and, more importantly, whether PD‐1 could be an auxiliary diagnostic biomarker for APPG.

## 2 MATERIALS AND METHODS

### 2.1 Study participants

A total of 205 eligible patients from The Third Affiliated Hospital of Guangzhou University of Chinese Medicine and Hai Zhu Qu Jiang Hai Jie Community Health Service Centre were enrolled in this study from 2018 to 2019. Patients were divided into four groups according to current gout classification standards: APPG (n = 36), NAPPG (n = 48), AH (asymptomatic hyperuricemia; n = 42) and NCs (normal controls; n = 79). Healthy patients were used as the NCs group. The collection of all clinical specimens was approved by the hospital clinical ethics committee. Written informed consent was obtained from each patient.

### 2.2 Diagnostic criteria for asymptomatic hyperuricemia

Patients were diagnosed with asymptomatic hyperuricemia according to the 2013 Consensus on the Treatment of Hyperuricemia and Gout Standards by Experts from the Endocrinology Branch of the Chinese Medical Association^28^: SUA levels of >420 μmol/L (7.0 mg/mL) in males and >360 μmol/L (6 mg/mL) in females with no patient specimens meeting the diagnostic criteria for gout.

### 2.3 Diagnostic criteria for PG

The T‐score for the type of joint affected, characteristics of the attack, disease onset, clinical evidence of gout, blood uric acid levels and related imaging examinations were scored according to the 2015 American Rheumatology Association/European Anti‐Rheumatic Alliance Gout Classification Standards.^5^ Patients with T‐scores ≥8 can be diagnosed as having PG. Patients with acute inflammation were classified as having APPG, while patients without acute inflammation were classified as having NAPPG.

### 2.4 Exclusion criteria

Patients were excluded from this study based on the following criteria^29^: (a) patients with PG were excluded if they had experienced pseudogout symptoms, including acute suppurative arthritis, rheumatoid arthritis, calcium dihydrogen pyrophosphate deposition, ankylosing spondylitis and psoriasis, or diseases such as arthritis, osteoarthritis and bone tumours; (b) patients with asymptomatic hyperuricemia were excluded if they had experienced other diseases that cause elevated blood uric acid levels, had taken drugs that can cause elevated blood uric acid levels and had a clear gout, a malignant tumour, and severe kidney disease diagnosis, or had insufficiency or severe systemic diseases (such as cachexia).

### 2.5 Specimen collection

All patients enrolled in this study were instructed to ingest no food or water for 12 hours to ensure that they had empty stomachs when their peripheral venous blood was collected in an EDTA anticoagulant tube early the next morning. The EDTA anticoagulated whole blood samples were used to isolate and extract PBMCs and detect PD‐1 mRNA expression.

### 2.6 PBMC isolation

Peripheral blood mononuclear cells were isolated within 6 hours of venous blood collection to ensure lymphocyte survival. The density gradient separation technique was used to isolate mononuclear cells from the blood at 18‐20°C using Ficoll‐Paque PLUS reagent according to the manufacturer's instructions.

### 2.7 Quantification of PD‐1 mRNA expression by qRT‐PCR

Total RNA was extracted from the isolated mononuclear cells using TRIzol reagent according to the manufacturer's instructions and reverse transcribed into cDNA using a TAKARA reverse transcription kit. PD‐1 mRNA expression levels in the PBMCs were detected using qRT‐PCR with 10 μmol/L of each primer under the following reaction conditions: 95°C for 5 minutes followed by 40 cycles of 95°C for 10 seconds, 60°C for 15 seconds and 72°C for 20 seconds. Each sample was tested three times independently. Data were processed using the ΔΔCT method^30^, ^31^: ΔΔCT = ΔCT unknown—ΔCT calibrator. The average relative content ($2^{‐\Delta \Delta C_{T}}$) was used for statistical analysis. The sequences of the primers used for qRT‐PCR are shown in Table S1.

### 2.8 Instruments and reagents

Ficoll‐PaqueTM PLUS reagent was purchased from Sweden, RNA extraction kits were purchased from Invitrogen (Karlsruhe, Germany), reverse transcription cDNA kits were purchased from Takara‐Bio (Tokyo, Japan), and PCR kits were purchased from Kangcheng Biotech (Shanghai, China). An Ultramicro Ultraviolet/visible Spectrophotometer was obtained from SimpliNano (New York, USA), a Hema9600 Gene Amplifier was obtained from Hema (Zhuhai, China), and a Rotor‐Gene Q qRT‐PCR analyser was obtained from Invitrogen.

### 2.9 Statistical analysis

Clinical data were analysed using two‐tailed independent *t* tests or ANOVA in GraphPad Prism (version 7.0). Between‐group comparisons were made using the Mann‐Whitney test or chi‐squared test, as appropriate. Pearson correlation analysis was performed to assess the correlations between PD‐1 mRNA and clinical variables using IBM SPSS Statistics 25 (SPSS Inc, Chicago, IL, USA). ROC curve analysis was performed to evaluate the diagnostic value of PD‐1 mRNA in PBMCs during different stages of PG. *P*‐value < 0.05 were considered statistically significant.

## 3 RESULTS AND DISCUSSION

PD‐1 expression in PBMCs has been associated with good clinical outcome in inflammatory diseases. Due to the increasing prevalence of PG and the lack of a useful diagnostic biomarker that can distinguish between different stages, we investigated whether PD‐1 is involved in APPG pathogenesis and, more importantly, whether PD‐1 could be an auxiliary diagnostic biomarker for APPG. In this study, we present data indicating the potential of PD‐1 mRNA as a biomarker associated with PG, with the strongest association with APPG, predictive value for the development of PG and progression of APPG.

**Figure 1:**
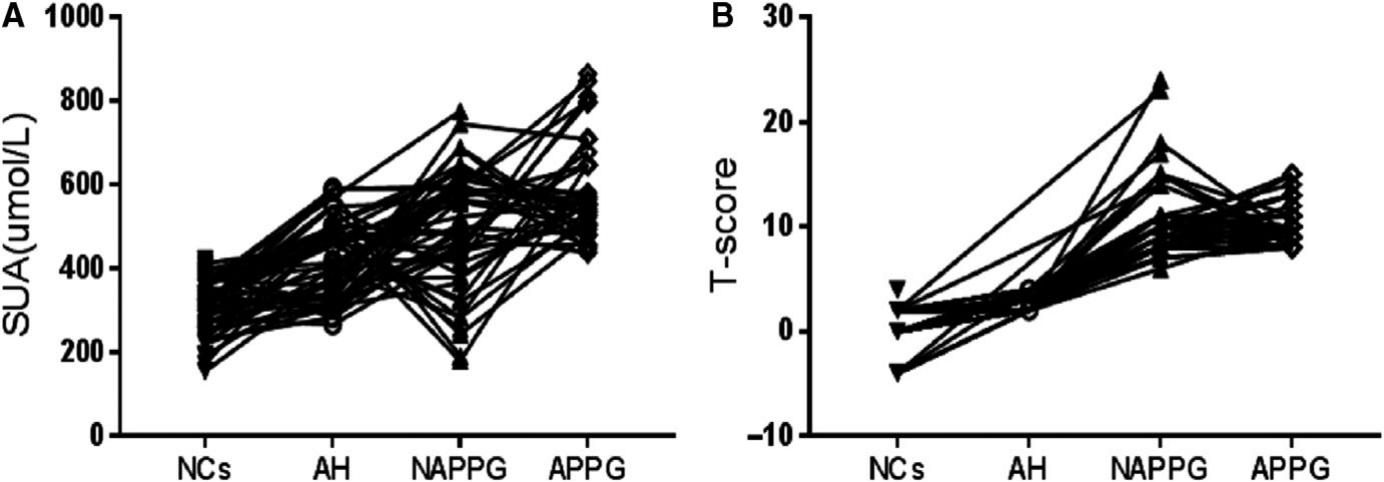
Serial measurements of SUA and T‐score. A, Serial measurements of SUA in serum samples obtained at different groups from 205 patients. B, Serial measurements of T‐score at different groups from 205 patients

First, we analysed the clinical data obtained from the patients included in this study. TG, Chol, FBG, SUA, WBC, lymphocyte and T‐score of APPG were significantly higher in patients with APPG than in NCs (*P* < 0.05), whereas FBG, WBC, lymphocyte and T‐score were significantly higher in patients with APPG than in patients with AH (*P* < 0.05). Moreover, TG, FBG, SUA and T‐score of patients with NAPPG were significantly higher than those in NCs (*P* < 0.05), whereas T‐score of patients with NAPPG was significantly higher than that of patients with AH (*P* < 0.01; Table S2). The majority of patients with NAPPG and APPG displayed characteristics of first metatarsophalangeal joint involvement at symptom onset, whereas those with NAPPG had typical recurrent attacks, and most of those with APPG experienced one typical attack. SUA levels were 0.48‐0.60 mmol/L in patients with NAPPG but were generally ≥0.60 mmol/L in those with APPG. Furthermore, urate deposition was observed in most joints or bursa in patients with NAPPG, but not in those with APPG (Table S3). The serum uric acid concentration in patients with primary gouty arthritis in the acute phase was high, but the T‐score did not fluctuate too much (Figure 1). The results showed that the typical recurrence of inflammation in patients with primary gouty arthritis in the acute phase was associated with elevated serum uric acid.

**Figure 2:**
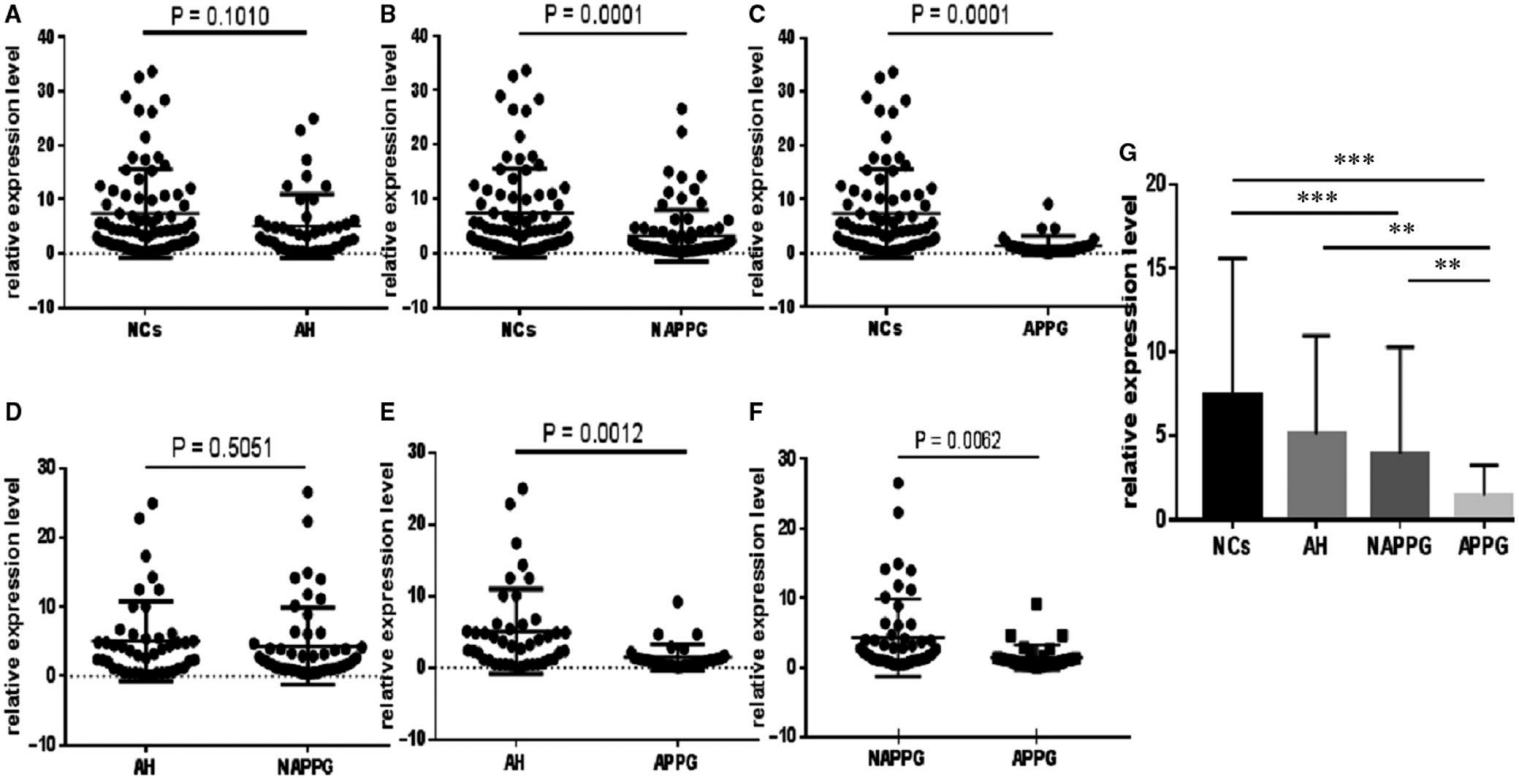
Differential analysis of PD‐1 mRNA expression in patients with PG and normal controls. qRT‐PCR assay results of PD‐1 mRNA expression. A‐F, Forest scatterplot: The qRT‐PCR assay was performed to verify the expression levels of PD‐1 mRNA in the NCs group, AH group, NAPPG group and APPG group. G, The histogram presents showed that PD‐1 mRNA expression levels progressively decreased from the NCs to patients with AH, NAPPG and APPG. **P < 0.01, ***P < 0.001

From a clinical point of view, we believe that the observed ability of PD‐1 mRNA to predict PG development is of key importance. We used qRT‐PCR to detect PD‐1 mRNA expression in the PBMCs of patients with different stages of PG. We observed that PD‐1 mRNA expression was significantly lower in patients with NAPPG and APPG than in NCs (*P* = 0.0001 and *P* = 0.0001, respectively). In addition, it was significantly lower in patients with APPG than in those with NAPPG (*P* = 0.0062). Thus, PD‐1 mRNA could be an auxiliary diagnostic biomarker for APPG. Moreover, PD‐1 mRNA expression levels progressively decreased from NCs to patients with AH, NAPPG and APPG and were significantly lower in patients with APPG than in those with NAPPG and AH and NCs. They were significantly lower in the NAPPG group than in the NC group (*P* < 0.01; Figure 2). These results indicate that PD‐1 mRNA expression is significantly down‐regulated in APPG. This effect may be due to malfunction and disorder of the secretory functions of T and B lymphocytes, resulting in decreased PD‐1 secretion, inhibition of T and B lymphocyte anti‐inflammatory responses and promotion of the pro‐inflammatory response mediated by T and B lymphocytes and cytokines. The subsequent imbalance between the anti‐ and pro‐inflammatory responses of T and B lymphocytes could result in local joint inflammation and worsened APPG. Indeed, studies have shown that PD‐1 mRNA expression in PBMCs reduces rheumatoid arthritis by causing T lymphocyte secretion dysfunction in these patients,^22^, ^32^ consistent with the findings of this study. Thus, PD‐1 mRNA may be involved in immune regulation during APPG.

We also analysed whether PD‐1 mRNA levels correlated with any of the clinical data and biochemical biomarkers in the study participants. Correlation analysis revealed no significant correlation between PD‐1 mRNA and height, weight, BMI, SBP, DBP, TG, Chol, FBG, SUA, WBC or lymphocytes, indicating that PD‐1 mRNA may not have an inflammation‐mediated relationship with these biomarkers. However, PD‐1 mRNA levels correlated negatively with T‐score (*r* = −0.209, *P* < 0.01; Table 1), which is the classification criterion for diagnosing patients with PG. These results indicate that PD‐1 mRNA is related to the clinical diagnosis of PG and could be used as an auxiliary diagnosis biomarker for PG.

**Table 1:** Pearson correlation analysis of clinical variables and PD‐1 mRNA expression

	Height	Weight	BMI	SBP	DBP	TG	Chol	FBG	WBC	Lymphocytes	SUA	T‐score	PD‐1 mRNA expression
Age	0.143*	−0.031	−0.102	0.001	−0.021	−0.017	0.091	0.243**	0.051	−0.121	−0.058	0.023	0.131
Height	—	0.102	−0.406**	−0.096	0.036	−0.092	−0.008	−0.019	−0.003	−0.042	0.066	−0.03	0.125
Weight		—	0.861**	0.117	0.166*	0.128	0.045	0.116	0.062	0.140*	0.085	0.327**	−0.03
BMI			—	0.158*	0.129	0.154*	0.041	0.106	0.055	0.144*	0.045	0.299**	−0.089
SBP				—	0.583**	0.157*	−0.055	0.170*	−0.047	0.066	0.08	0.146*	0.054
DBP					—	0.137*	−0.023	0.068	0.026	0.157*	0.237**	0.232**	−0.025
TG						—	0.123	0.289**	0.03	0.017	0.227**	0.380**	−0.104
TC							—	0.074	0.065	0.006	0.018	0.160*	−0.023
GLU								—	0.033	0.018	0.103	0.433**	0.016
WBC									—	0.005	−0.005	0.061	−0.014
Lymphocytes										—	0.017	0.119	−0.044
SUA											—	0.304**	−0.081
T‐score												—	−0.209**

Abbreviations: BMI, body mass index; Chol, cholesterol; DBP, diastolic blood pressure; FBG, fasting blood glucose; SBP, systolic blood pressure; SUA, serum uric acid; TG, triglyceride; WBC, white blood cell count.

* *P* < 0.05.

** *P* < 0.01.

**Figure 3:**
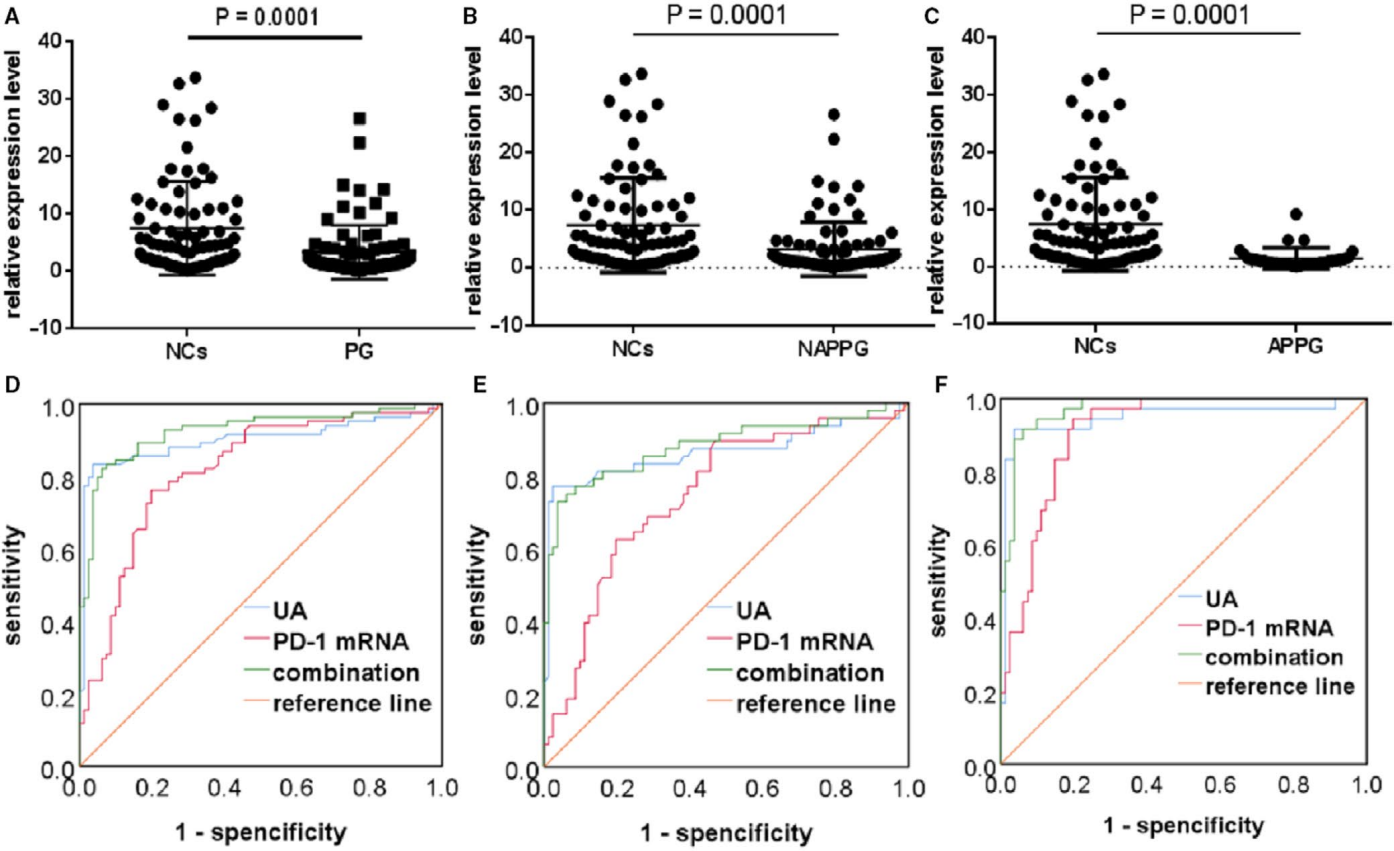
Diagnostic performance of PD‐1 mRNA in different stages of PG compared to healthy controls. A‐C, Forest scatterplot: The qRT‐PCR assay was performed to verify the expression levels of PD‐1 mRNA in the NCs group, PG group, NAPPG group and APPG group. D‐F, ROC curve analysis of SUA, PD‐1 mRNA and the combination of both biomarkers. PG: NAPPG + APPG

Finally, we evaluated whether PD‐1 mRNA could be an alternative to SUA as a biomarker for PG. Therefore, we performed ROC curve analysis to determine the diagnostic value of PD‐1 mRNA levels in patients with different stages of PG. PD‐1 mRNA expression was significantly lower in the PG group than in the NC group (*P* < 0.001; Figure 3A), with ROC curve analysis showing that SUA (AUC: 0.900, *P* < 0.05) has a better diagnostic performance than PD‐1 mRNA (AUC: 0.818, *P* < 0.05), whereas the combination of both biomarkers displayed the best diagnostic performance for PG (AUC: 0.925, *P* < 0.05; Figure 3D). PD‐1 mRNA expression was also significantly lower in the NAPPG group than in the NCs group (*P* < 0.001; Figure 3B), with ROC curve analysis revealing that SUA (AUC: 0.865, *P* < 0.05) had a better diagnostic performance than PD‐1 mRNA (AUC: 0.749, *P* < 0.05) but their combination had the best diagnostic performance for NAPPG (AUC: 0.882, *P* < 0.05; Figure 3E). In addition, PD‐1 mRNA levels were significantly lower in the APPG group than in the AH group (*P* < 0.001; Figure 3C), with ROC curve analysis revealing that SUA (AUC: 0.947, *P* < 0.05) displayed better diagnostic performance than PD‐1 mRNA (AUC: 0.910, *P* < 0.05), whereas the combination of both biomarkers had the best diagnostic performance for APPG (AUC: 0.972, *P* < 0.05; Figure 3F). These results suggest that both SUA and PD‐1 mRNA can be used as auxiliary diagnostic biomarkers for patients with different stages of PG, but that SUA is more effective than PD‐1 mRNA when diagnosing different stages of PG. Moreover, the diagnostic performance was better in patients with APPG.

**Figure 4:**
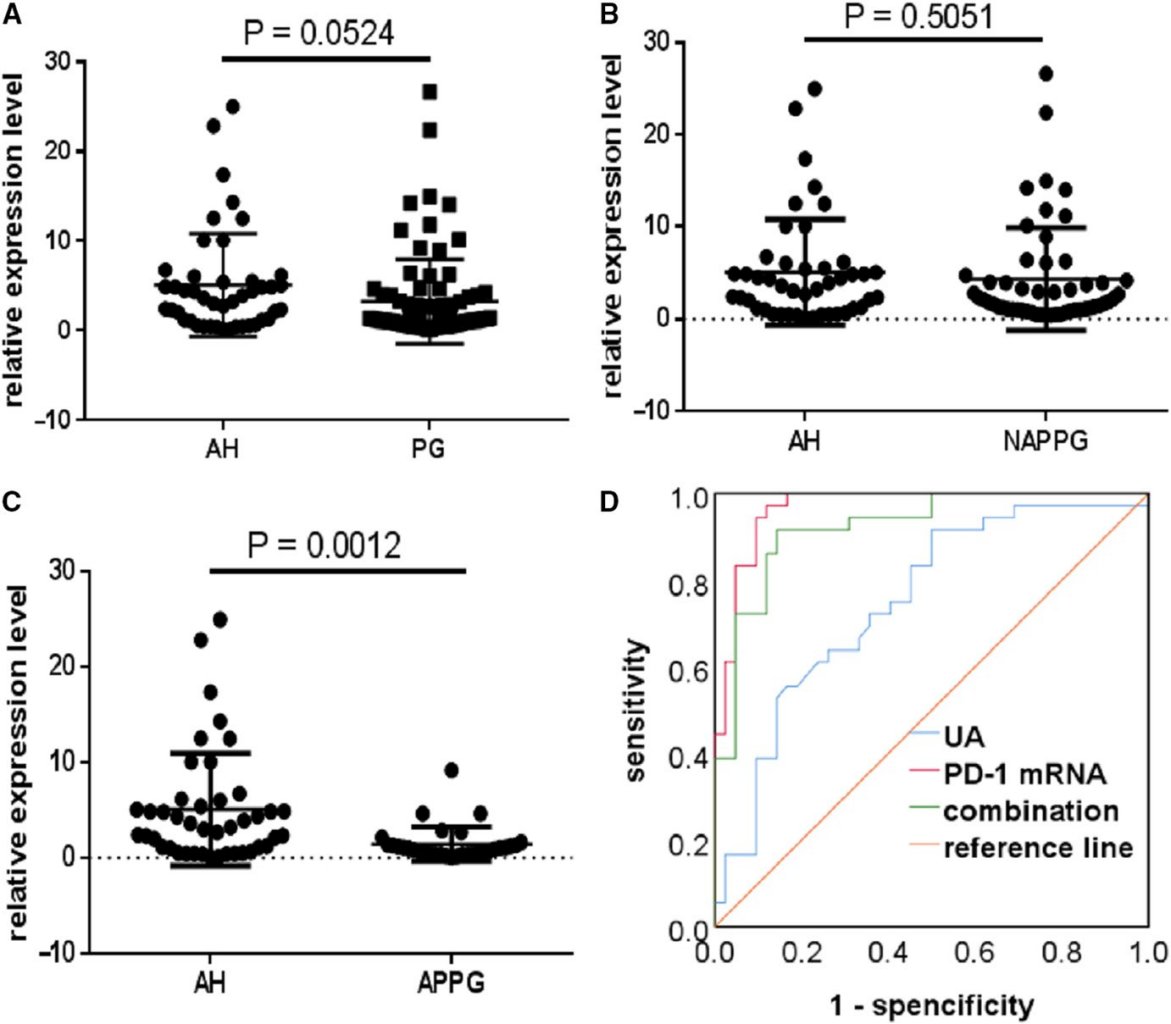
Diagnostic performance of PD‐1 mRNA in different stages of PG compared to asymptomatic hyperuricemia (AH). A‐C, Forest scatterplot: The qRT‐PCR assay was performed to verify the expression levels of PD‐1 mRNA in the AH group, PG group, NAPPG group and APPG group. D, ROC curve analysis of SUA, PD‐1 mRNA and the combination of both biomarkers. PG: NAPPG + APPG

Next, we compared the diagnostic value of PD‐1 mRNA levels when differentiating between patients with different PG stages and those with asymptomatic hyperuricemia. We evaluated whether the AUC of the ROC analysis differs between the two biomarkers. PD‐1 mRNA expression was significantly lower in the APPG group than in the AH group (*P* = 0.0012; Figure 4C). ROC curve analysis showed that PD‐1 mRNA (AUC: 0.967, *P* < 0.05) had better diagnostic performance than SUA (AUC: 0.749, *P* > 0.05) and the two markers combined (AUC: 0.923, *P* < 0.05; Figure 4D) for APPG. Thus, these results suggest that while both SUA and PD‐1 mRNA can be used as auxiliary diagnostic biomarkers for APPG, PD‐1 mRNA has better diagnostic performance than SUA. Since SUA levels change during each stage of PG, they cannot be used to diagnose patients with different stages of PG, and also as there was a significant overlap in SUA levels between different stages of PG, there is only a limited value for SUA as a diagnostic biomarker. Therefore, we recommend PD‐1 mRNA as an auxiliary biomarker for the diagnosis of APPG.

**Figure 5:**
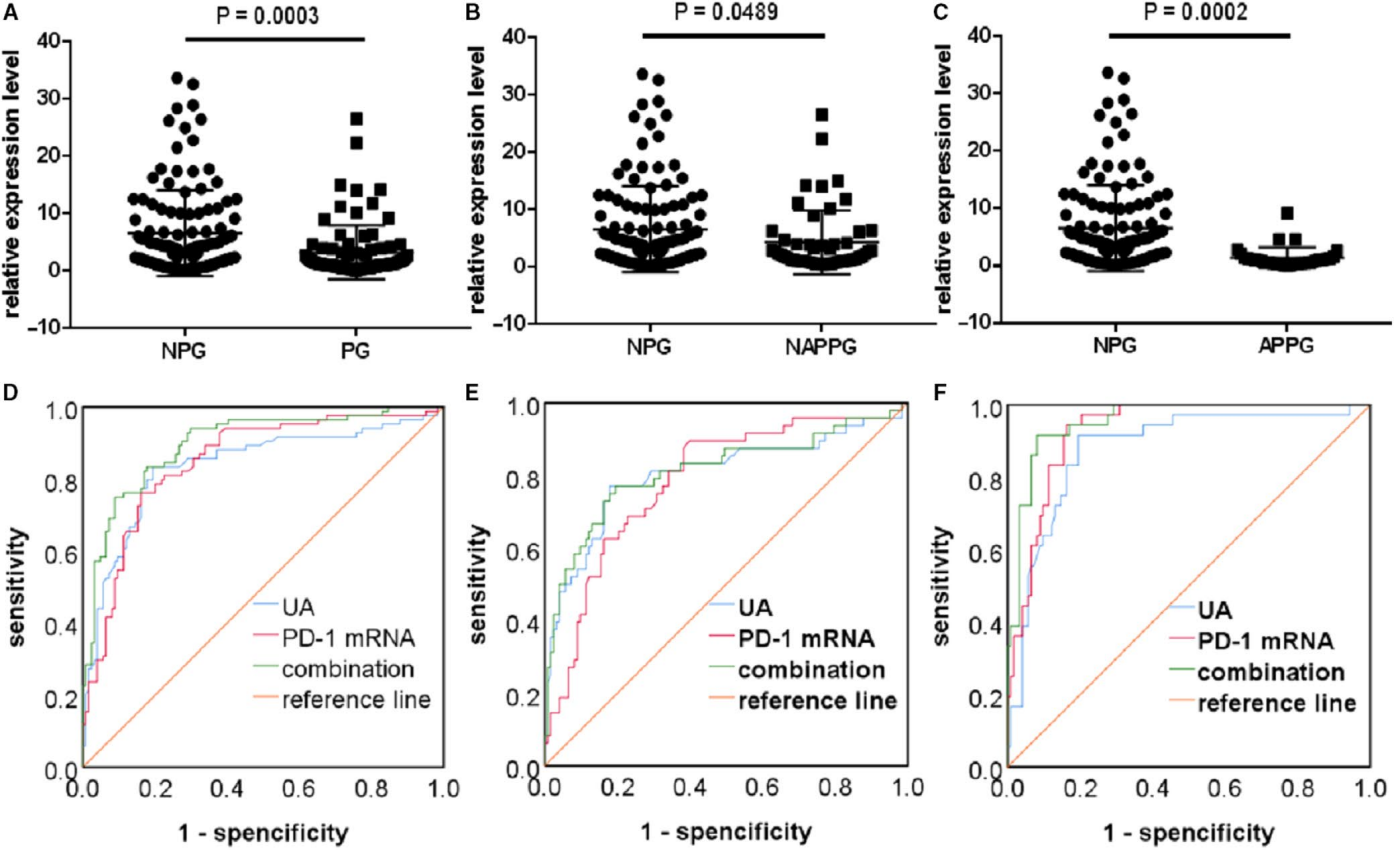
Diagnostic performance of PD‐1 mRNA in different stages of PG compared to patients with non‐PG. A‐C, Forest scatterplot: The qRT‐PCR assay was performed to verify the expression levels of PD‐1 mRNA in the NPG group, PG group, NAPPG group and APPG group. D‐F, ROC curve analysis of SUA and PD‐1 mRNA and the combination of both biomarkers. NPG: NCs + AH; PG: NAPPG + APPG

Finally, we compared PD‐1 mRNA expression levels in patients with non‐PG and those with different stages of PG. PD‐1 mRNA levels were significantly lower in the PG group than in the NPG group (*P* = 0.0003; Figure 5A), with ROC curve analysis showing that PD‐1 mRNA (AUC: 0.848, *P* < 0.05) has better diagnostic performance than SUA (AUC: 0.837, *P* < 0.05), while their combination (AUC: 0.897, *P* < 0.05) had even better diagnostic performance for PG (Figure 5D). These findings suggest that PD‐1 mRNA is a promising biomarker for the differential diagnosis of non‐PG and different stages of PG. In addition, PD‐1 mRNA expression was lower in the NAPPG group than in the NPG group (*P* = 0.0489; Figure 5B), with ROC curve analysis revealing that PD‐1 mRNA (AUC: 0.785, *P* < 0.05) displayed worse diagnostic performance than SUA (AUC: 0.804, *P* < 0.05) for NAPPG, while their combination displayed the best diagnostic performance (AUC: 0.812, *P* < 0.05) (Figure 5E). These findings indicate that compared to SUA, PD‐1 mRNA is less able to distinguish between patients with non‐PG and those with NAPPG. Moreover, PD‐1 mRNA levels were significantly lower in the APPG group than in the NPG group (*P* = 0.0002; Figure 5C), with ROC curve analysis showing that PD‐1 mRNA (AUC: 0.930, *P* < 0.05) has better diagnostic performance than SUA (AUC: 0.880, *P* < 0.05) for APPG, while their combination (AUC: 0.955, *P* < 0.05) displayed even better diagnostic performance (Figure 5F). These findings suggest that PD‐1 mRNA performs best as an auxiliary diagnostic biomarker for APPG and that PD‐1 mRNA expression can differentiate between the diagnosis of asymptomatic hyperuricemia and APPG. Since SUA levels cannot differentiate between patients with different stages of PG, PD‐1 mRNA can be recommended as a biomarker for the auxiliary diagnosis of APPG. Thus, PD‐1 mRNA could have a significant impact on the diagnosis of PG and provide new directions for the development of novel drugs to treat PG.

Despite these significant findings, this study has some limitations. First, the sample size used in this study was relatively small; thus, our experimental data require verification by large‐scale, multi‐centre and multiethnic studies. Second, we cannot exclude the possibility that patients with PG were incorrectly classified, which would affect our conclusion that PD‐1 mRNA can be used as an auxiliary diagnostic biomarker of APPG, or that other underlying diseases and genetic factors affected the outcome in these patients. Third, we used no other methods to verify PD‐1 levels, such as flow cytometry to detect PD‐1, ELISA to detect serum sPD‐1 levels or Western blotting to detect PD‐1 expression. However, since the qRT‐PCR method has previously been used to identify auxiliary diagnostic biomarkers^33^, ^34^ successfully, we are confident that our methods are adequate for this preliminary study.

Importantly, the current study has significant strengths. This study is the first to examine the relationship between PD‐1 mRNA and PG and is the first to identify that PD‐1 mRNA expression levels were lower in the PBMCs of patients with APPG, thus could be used as an auxiliary diagnostic biomarker for APPG. Moreover, the combination of PD‐1 mRNA and SUA is more effective than SUA alone for the clinical diagnosis of APPG. Therefore, this study provides a theoretical and experimental basis for the study of novel immune targets for treating PG.

## CONFLICT OF INTEREST

The authors disclose no conflict of interests.

## AUTHOR CONTRIBUTIONS

**Zhao Kewei:** Conceptualization (equal); funding acquisition (equal); project administration (equal); resources (equal). **Jing Su:** Data curation (equal); formal analysis (equal); investigation (equal); methodology (equal); software (equal); writing‐original draft (equal); writing‐review & editing (equal). **Xuefang Zhang:** Data curation (equal); formal analysis (equal); writing‐review & editing (equal). **Qing Zhao:** Data curation (equal); formal analysis (equal); investigation (equal); writing‐review & editing (equal). **Zhaodi Guo:** Investigation (equal); methodology (equal); writing‐review & editing (equal). **Jianxiong Wu:** Investigation (equal); writing‐review & editing (equal). **Guoqiang Chen:** Investigation (equal); writing‐review & editing (equal). **Qianxin Liang:** Formal analysis (equal); writing‐review & editing (equal). **Zhixiang Chen:** Methodology (equal); writing‐review & editing (equal). **Zhiliang He:** Formal analysis (equal); writing‐review & editing (equal). **Xiuping Cai:** Methodology (equal); writing‐review & editing (equal). **Manlin Xie:** Formal analysis (equal); writing‐review & editing (equal). **Lei Zheng:** Conceptualization (equal); project administration (equal); writing‐review & editing (equal).

## Supporting information

Supplementary Material

## Data Availability

The data that support the findings of this study are available from the corresponding author upon reasonable request.
