# Supplementary material for: PD‐1 mRNA expression in peripheral blood mononuclear cells as a biomarker for different stages of primary gouty arthritis
Source: J Cell Mol Med. 2020 Jul 8;24(16):9323–31. doi: 10.1111/jcmm.15582 (PMC7417685; doi:10.1111/jcmm.15582)
Supplement: Supplementary file 1 — Supplementary Material [file JCMM-24-9323-s001.doc]

**Supplementary Material**

**Table S1. qRT-PCR primer sequences**

|  | 5'-3' (Forward) | 5'-3' (Reverse) | Product length (bp) |
| --- | --- | --- | --- |
| Human β-actin | TGACGTGGACATCCGCAAAG | CTGGAAGGTGGACAGCGAGG | 161 |
| PD-1 | CCAGGATGGTTCTTAGACTCCC | TTTAGCACGAAGCTCTCCGAT | 145 |

**Table S2.** Statistical analysis of the clinical variables of the patients included in this study

| Clinical variables | NCs (*n* = 79) | AH (*n* = 42) | NAPPG (*n* = 48) | APPG (*n* = 36) |
| --- | --- | --- | --- | --- |
| Male, *n* | 50 | 30 | 35 | 20 |
| Age (years) | 52.76 ± 1.315 | 60.17 ± 2.816 | 57.13 ± 2.04 | 51.36 ± 2.873 |
| Height (m) | 1.683 ± 0.008 | 1.668 ± 0.019 | 1.667 ± 0.008 | 1.674 ± 0.001 |
| Weight (kg) | 60.47 ± 1.38 | 63.28 ± 1.642 | 71.29 ± 1.895 | 68.82 ± 2.052 |
| BMI (kg/m^2^) | 21.42 ± 0.508 | 23.09 ± 0.776 | 25.75 ± 0.751 | 24.61 ± 0.772 |
| SBP (mmHg) | 130.5 ± 2.264 | 136 ± 2.862 | 139 ± 2.843 | 134.8 ± 2.755 |
| DBP (mmHg) | 76.2 ± 1.073 | 75.1 ± 1.469 | 79.27 ± 1.811 | 85.39 ± 2.237 |
| TG (mmol/L) | 1.271 ± 0.1075 | 1.895 ± 0.1671 | 2.238 ± 0.1951* | 2.414 ± 0.2782* |
| Chol (mmol/L) | 4.71 ± 0.1383 | 5.626 ± 0.2231 | 4.798 ± 0.2347 | 6.461 ± 1.193* |
| FBG (mmol/L) | 5.288 ± 0.08 | 5.75 ± 0.1042 | 6.347 ± 0.26716* | 6.357 ± 0.3158*# |
| SUA (umol/L) | 312.2 ± 8.169 | 500.6 ± 10.06 | 509.4 ± 22.5** | 521.6 ± 19.39** |
| WBC (^10^9^/L) | 6.177 ± 0.1898 | 6.673 ± 0.2812 | 6.937 ± 0.2632 | 8.319 ± 0.4526**## |
| Lymphocyte (^10^9^/L) | 2.196 ± 0.1201 | 2.213 ± 0.1042 | 3.722 ± 0.9445 | 5.183 ± 2.94**## |
| T-score | 0.1519 ± 0.2438 | 2.738 ± 0.1183 | 10.69 ± 0.5548**## | 10.03 ± 0.3415**## |

*comparison with NCs, **P* < 0.05, ***P* < 0.01; #comparison with AH, #*P* < 0.05, ##*P* < 0.01.

NCs: normal controls; AH: asymptomatic hyperuricemia; NAPPG: non-acute phase of primary gouty arthritis; APPG: acute phase of primary gouty arthritis.

BMI: body mass index; SBP: systolic blood pressure; DBP: diastolic blood pressure; TG: triglyceride; Chol: cholesterol; FBG: fasting blood glucose; SUA: serum uric acid; WBC: white blood cell count.

**Table S3. Gout diagnosis score breakdown of the study participants**

| Gout diagnosis points | Category | Grade | NCs | AH | NAPPG | APPG |
| --- | --- | --- | --- | --- | --- | --- |
| 1. Types of joints affected at the onset of symptoms | Ankle or midfoot (not first metatarsophalangeal joint) | 1 | 0 | 0 | 10 | 10 |
|  | First metatarsophalangeal joint involvement | 2 | 0 | 0 | 38 | 26 |
| 2. Features of symptom onset | Affected joints are red | 1 | 0 | 0 | 9 | 1 |
|  | Affected joints cannot tolerate touch or compression | 2 | 0 | 0 | 9 | 2 |
|  | Difficulty walking or moving the affected joints | 3 | 0 | 0 | 30 | 33 |
| 3. Time course of symptoms. At least two symptoms with or without anti-inflammatory treatment:  (1) Pain peaks within 24 hours  (2) Symptoms are relieved within 14 days  (3) Inter-seizure symptoms completely relieved | One typical attack | 1 | 0 | 0 | 36 | 3 |
|  | Recurrent typical seizures | 2 | 0 | 0 | 12 | 33 |
| 4. Clinical evidence of goutstones | Dry or chalky subcutaneous nodules under the transparent skin, usually overlying blood vessels at joints, ears, bursa, digits, and tendons | 4 | 0 | 0 | 0 | 7 |
| 5. Blood uric acid detected using the urase method | < 4 mg/dL (< 0.24 mmol/L) | -4 | 14 | 0 | 0 | 0 |
|  | 4-6 mg/dL (0.24-0.36 mmol/L) | 0 | 31 | 0 | 0 | 0 |
|  | 6-8 mg/dL (0.36-0.48 mmol/L) | 2 | 34 | 19 | 0 | 7 |
|  | 8-10 mg/dL (0.48-0.60 mmol/L) | 3 | 0 | 15 | 28 | 10 |
|  | ≥ 10 mg/dL (≥ 0.60 mmol/L) | 4 | 0 | 8 | 20 | 19 |
| 6. Analysis of synovial fluid with symptoms | MSU negative | -2 | 0 |  | 0 | 0 |
| 7. Imaging evidence of urate deposition in joints or bursa where symptoms have occurred | Ultrasound confirmed "bilateral sign" or DECT confirmed urate deposition | 4 | 0 | 0 | 14 | 0 |
| 8. Imaging evidence of gout-related joint injuries | X-rays confirm erosion of at least one joint in the hands and/or feet | 4 | 0 | 0 | 6 | 2 |

MSU: monosodium urate; DECT: dual-source CT; *no relevant test results or uric acid levels ≥ 4 and < 6 mg/dl (≥ 0.24 and < 0.36 mmol/L). The score is 0, with a total score of ≥ 8 needed to diagnose gout.

NCs: normal controls; AH: asymptomatic hyperuricemia; NAPPG: non-acute phase of primary gouty arthritis; APPG: acute phase of primary gouty arthritis.
